# Angiotensin-(1–7) oral formulation improves physical performance in mountain bike athletes: a double‐blinded crossover study

**DOI:** 10.1186/s13102-021-00274-4

**Published:** 2021-05-06

**Authors:** Samara Silva de Moura, Adália Táci Pereira Mendes, Francisco de Assis Dias Martins-Júnior, Nádia Lúcia Totou, Daniel Barbosa Coelho, Emerson Cruz de Oliveira, Daisy Motta-Santos, Robson Augusto Souza dos Santos, Lenice Kappes Becker

**Affiliations:** 1 Postgraduate Program in Health and Nutrition/PPGSN, Federal University of Ouro Preto, Ouro Preto, Brazil; 2 Physical Education School, Federal University of Ouro Preto, Ouro Preto, Brazil; 3 Postgraduate Program in Biological Sciences, Institute of Exact and Biological Sciences, Federal University of Ouro Preto, Ouro Preto, Brazil; 4 Department of Sports, School of Physical Education, Physiotherapy and Occupational Therapy, Federal University of Minas Gerais, Belo Horizonte, Brazil; 5 Department of Physiology and Biophysics, Institute of Biological Sciences, Federal University of Minas Gerais, Belo Horizonte, Brazil

**Keywords:** Exercise, Performance, Nutrition, cardiovascular/cardiorespiratory

## Abstract

**Background:**

The ECA2/Ang-(1–7)/Mas axis is shown to be involved in effects mediated by physical exercise, as it can induce the release of nitric oxide (ON) and bradykinin (BK), which are potent vasodilators. The vasodilating action the NO/BK can contribute to increased metabolic efficiency in muscle tissue and central nervous system. The formulation HPβ-CD-Ang-(1–7) through its mechanisms of action can be a promising supplement to aid in the maintenance and improvement of performance and may also favor recovery during competitions. The premise of this study was to investigate the effects of acute oral supplementation HPβ-CD-Ang-(1–7) on the performance of mountain bike (MTB) practitioners.

**Methods:**

Fourteen recreational athletes, involved in training programs for at least one year, participated in this crossover design study. Subjects underwent two days of testing with a seven-day interval. HPβ-CD-Ang-(1–7) (1.75 mg) and HPβCD-Placebo were provided in capsules three hours prior to tests. To determine the safety of the HPβ-CD-Ang-(1–7) formulation associated with physical effort, cardiovascular parameters heart rate (HR) and blood pressure (BP) were analyzed. Physical performance was measured using maximal oxygen uptake (VO_2_), total exercise time (TET), mechanical work (MW), mechanical efficiency (ME), and rating of perceived exertion (RPE). Respiratory exchange coefficient (REC), lactate and non-esterified fatty acids (NEFAs) were measured. Maximal incremental tests were performed on a progressively loaded leg cycle ergometer.

**Results:**

There were no significant differences in terms of HR or BP at rest and maximum effort between the HPβ-CD-Ang-(1–7) and placebo groups. The VO_2_max showed significant differences (*p* = 0.04). It was higher in the Ang-(1–7)condition (66.15 mlO_2_.kg^− 1^.min^− 1^) compared to the placebo (60.72 mlO_2_.kg^− 1^.min^− 1^). This was also observed for TET (Ang-(1–7) 39.10 min vs. placebo 38.14 min; *p* = 0.04), MW (Ang-(1–7) 156.7 vs. placebo 148.2; *p* = 0.04), and at the lowest RPE (Ang-(1–7) vs. placebo; *p* = 0.009). No significant differences were observed for REC, NEFAs, or Lactate.

**Conclusions:**

These results suggest that HPβ-CD-Ang-(1–7) improves the physical performance of MTB recreational athletes and could be a promising supplement.

**Trial registration:**

RBR-2 × 56pw8, registered January 15th, 2021. The study was prospectively registered.

## Background

The renin-angiotensin system (RAS) recognized as a system involved in cardiovascular and electrolyte control [1], plays a key role in various other physiological systems. The skeletal muscle is one of them [2, 3] the RAS system has important effects in skeletal muscle that can affect the physical performance, as well insulin sensitivity improve [4] and muscle atrophy inhibition [5].

The heptapeptide the Angiotensin-(1–7), is traditionally recognized by cardiovascular effects [6], but studies show the influence in performing physical exercises. In a study conducted by Motta-Santos [7], Knockout mice by the angiotensin II converting enzyme (ECA2), an enzyme responsible for the production of angiotensin-(1–7) from Ang II, showed less physical performance and less cardiac adaptation to exercise[7].

Latest data from our laboratory have shown, that treatment with the oral formulation of Ang-(1–7) included in HPβ-CD, a cyclodextrin used in the formation of drug inclusion compounds that improves stability, solubility, bioavailability, uniform activation, absorption and gastric protection [8] prevents exercise-induced muscle damage in young people undergoing a protocol of injury induced by physical exertion (i.e., eccentric exercise). This formulation was associated with lower perception of acute muscle pain, as well as improvement in maximum strength levels and lower levels of proinflammatory cytokines at 48 and 72 h after the exercise session [9]. This suggests that the formulation HPβCD-Ang-(1–7) attenuates muscle damage in addition to maintaining physical performance showed by improve in strength levels [9].

Another important effect of Ang-(1–7) is the augments the bioavailability of nitric oxide (NO), promoting increased expression and activation of endothelial nitric oxide synthase (eNOS) via the Akt (PKB) protein-dependent signaling pathway, inducing vasodilation [10, 11]. Seeing that NO is an important mediator of several physiological processes, regulating tissue blood flow, muscle contraction, and mitochondrial biogenesis, Ang-(1–7) may act through this molecule, affecting positively physical performance [12].

Evidence indicates that, in addition to vasodilatory effects, transgenic rats with high levels of circulating Ang-(1–7) showed better tolerance and insulin-stimulated glucose uptake [13]. Another study [14] demonstrated increased muscle microvascular recruitment following Ang-(1–7) infusion, increasing glucose uptake via the Glut-4 receptor, this date indicate that this peptide affect the muscle metabolic control.

In central nervous system the XIE; ZHU; JI; TIAN et al. [15] the Ang-(1–7) reduced the cognitive deficits; this neuroprotective effect was associated with increased NO generation. Beyond neuroprotection, the Ang-(1–7) promotes a reduction in the release of norepinephrine monoamine (NA) centrally by BK / NO dependent manner [16, 17]. The central fatigue during exercise is associate with increase in NA and other monoamines (serotonin and dopamine) [18], so it is possible that the Ang-(1–7) contribute with reduction of the central fatigue during exercise.

Based on the evidence of both animal and human models regarding the physiological effects of Ang-(1–7), the present study aims to evaluate the effects of the HPβ-CD-Ang-(1–7) oral formulation on the physical performance of MTB athletes. In addition, considering the effects of Ang-(1–7) on cardiovascular and central nervous system, we evaluated the blood pressure, heart rate and perceived effort responses after exercise associate to supplement Ang-(1–7) orally.

## Methods

### Ethical aspects

This was an experimental study conducted at the Physical Education School, Federal University of Ouro Preto in the Exercise Physiology Laboratory, approved by the Human Research Ethics Committee under protocol no. 25402813.2.1001.5150. We submitted the present study to the Brazilian Registry of Clinical Trials (ReBEC), and it was approved under number RBR-2 × 56pw8, registered January 15th, 2021. To participate in the study, the participants were made aware of the study objectives and the possible benefits and risks. All provided informed written consent. This study adheres to CONSORT guidelines [19].

### Sample calculation

The sample size was calculated based on a previous study by our research group [9], that used the same dosage of HPβ-CD-Ang-(1–7) in individuals submitted to eccentric exercise protocol, we used the biochemical marker creatine kinase (CK) for the sample calculation. The analysis were performed using the Bioestat software (5.3), the CK showed a variation of 197 U / L ± 159U / L in the study, the statistical power was of 0.8, and a significance level of 0.05, the sample calculation resulted in 11 volunteers per group.

### Eligibility criteria

The inclusion criteria were involvement in cycling training programs for at least 12 months and carrying out at least four MTB training sessions per week.

Participants were excluded if they had used supplements with a potential stimulatory effect on the cardiovascular system (such as caffeine and guarana powder). Has had current injuries or in the past six months, has not been willing to abstain from intense exercise 24 h before the test. Participants were tested at the same time of day for each of the experimental visits.

### Recruitment

The first author of the study performed the interventions and recruited the volunteers. Participants were recruited in local community from June to August 2016. The data assessment started in August 2016 and ended in October 2016, after reaching the estimated number of participants.

### Sample characteristics

**Fig. 1:**
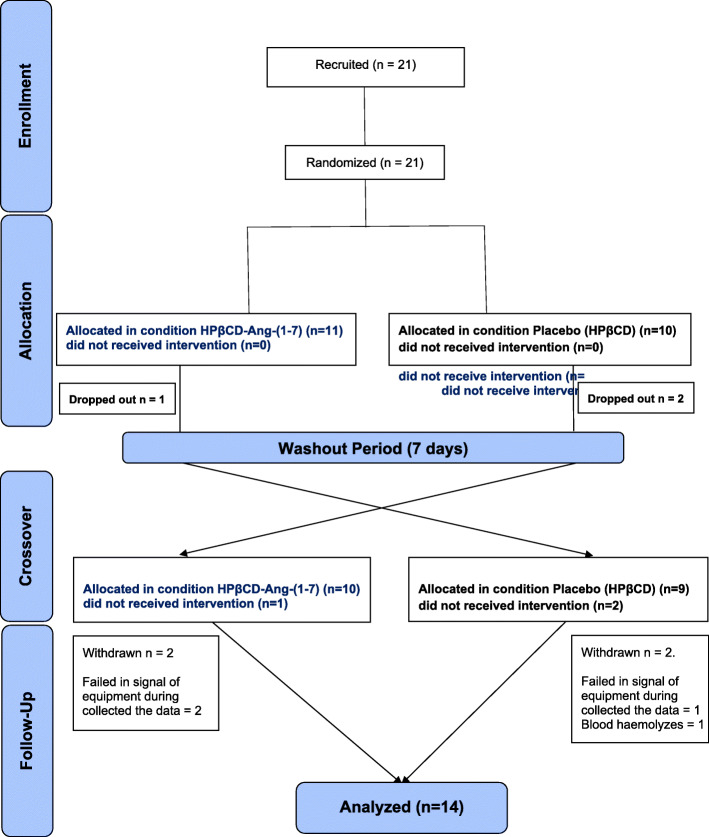
CONSORT flow diagram

Altogether, 21 cyclists of both sex (3 female and 18 male), volunteered for this study. For the final analysis, 14 volunteers (2 female and 12 male) were considered eligible (see CONSORT flow diagram, Fig. 1). Average age was 29 ± 5 years; Average body weight was 71 ± 7 kg; Average height was 1.70 ± 0.07 m; Average body mass index was 24 ± 2 kg/m^2^.

### Experimental design

The distribution of the formulation was double-blind and randomized. Participants received the formulation of HPβ-CD-Placebo or HPβ-CD-Ang-(1–7) (1.75 mg), 50 % of subjects randomly used HPβ-CD-Placebo in the first session and HPβ-CD-Ang-(1–7) in the second session or vice versa. The double blind and the randomization of the experiments were performed by the corresponding researcher. The same, assigned a code for each participant. The code was revealed to other researchers only at the time of data interpretation.

A single dose of the HPβ-CD-Placebo or formulation HPβ-CD-Ang-(1–7) was given orally, both in capsule form, three hours before the beginning of each test. The capsules were identical in color, size and without flavor, ensuring the blinding of the participants.

**Fig. 2:**
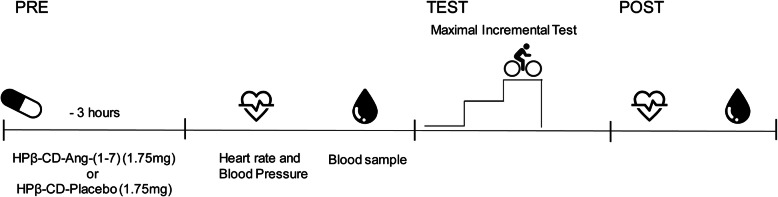
Experimental trial schematic. Participants completed both conditions in a double-blind, randomized, crossover manner, each separated by ≥ 7 days

During the intervention, we requested that physical training, food intake, and sleep hours be maintained. The volunteers were subjected to two days of tests with a seven-day interval between them, which can be considered a safe washout time. Upon return to the laboratory, we ask from the subjects to determine if there were adverse reactions and if they had maintained their diet and physical training routines. A schematic for the experimental trial is shown in Fig. 2.

### Physical exercise protocol using the leg ergometer cycle

The test protocol was based on previous studies with MTB athletes [20]. The tests were performed using a leg ergometer cycle (Biotec 2100, CEFISE Biotechnology), with 10 min of warm up and a load of 12.5 W for both genders. After warming up, a continuous progressive load test was performed with an initial load of 25 W for women and 37.5 W for men. The load was increased by 12.5 W every three minutes for both genders. The test finished when the individual reached voluntary exhaustion or when the rotation could not be kept at 70 rpm. At the end of each stage, heart rate (HR), ratings of perceived exertion (RPE), maximal oxygen uptake (VO_2_), and respiratory exchange coefficient (REC) were collected.

### Supplementation protocol

The formulation was developed by the Laboratory of Hypertension and Laboratory of Chemical of the University of Federal of Minas Gerais; the details were described previously [21]. This compound (HPβCD/Ang-(1–7)) was patented (BR 10 2016 0244064/Federal University of Minas Gerais/Federal University of Ouro Preto).

Oral supplementation with HPβCD-angiotensin-(1–7) (1.75 mg) or HPβCD-placebo (1.75 mg) was administered 3 h before the test protocol. The 3 h time was pre-established considering the peak action of angiotensin-(1–7) which has a window of action between 2 and 6 h [22].

Considering toxicity and adverse responses in humans, the present study used a dose 16 times lower (1.75 mg) than the dose used in a study conducted in cancer patients (400 µg/kg)[23] that showed no collateral effects. In addition, a previous study in healthy younger [9] showed protective effects against muscle damage using the same dose without side-effects.

### Plasma analysis

Blood samples were collected from the antecubital vein by a skilled phlebotomist using standard technical venipuncture. Approximately 12 ml were collected in vacutainer tubes. Immediately after collection, the blood was centrifuged at 3.000 rpm for ten minutes, and the serum transferred to Eppendorf tubes stored at − 80 °C. Aliquots were used to measure non-esterified fatty acids (NEFA) and creatine kinase (CK).

### Blood lactate levels

Lactate levels were measured in each participant’s index finger by placing one drop (5 µl) of blood on a BM-Lactate reagent strip (Roche Diagnostics GmbH, D68298 Mannheim, Germany) and introducing it into the Accutrend® Lactate meter (Roche Diagnostics GmbH, D-68,298 Mannheim, Germany). Lactate was measured immediately at the end of each test.

### Non‐esterified fatty acids (NEFAs)

NEFAs were analyzed according to the specific colorimetric method using the Randox® kit (Randox Laboratories, Oceanside, CA, USA).

### Mechanical work and efficiency

To calculate mechanical work and efficiency, the equations above were used [24]. Work is described as follows: *w = time (min) × load (kg) × wheel circumference (m) × rotation (rpm)*, in kg.m., while mechanical efficiency was calculated using the equation:

$$mechanical efficiency= \frac{useful work \left(\frac{mechanical work \left(kg.m\right)}{perfect machineconstant \left(kg.m\right)}\right)}{energy expenditure \left(kcal\right)} \times 100$$,

where the perfect machine constant is the energy spent by a machine without loss of efficiency to perform work (1 kcal = 426.4 kg.m) in %.

### Evaluation of BP and HR

Blood pressure was measured with an aneroid sphygmomanometer (Missouri®, Brazil) and stethoscope (Missouri®, Brazil) before and immediately after (20 s) the end of the test protocol. HR was measured with Polar RS800 (POLAR, Finland) at rest, along the steps and at the peak of maximum physical test effort.

### Evaluation of subjective rating of perceived exertion (RPE)

Participants evaluated their fatigue using the subjective rating of perceived exertion with reference to the Borg Scale [25]. RPE was determined at each stage completed by the volunteer.

### Evaluation of maximum oxygen consumption

The aerobic capacity was determined in the HPβ-CD-placebo and HPβ-CD-Ang-(1–7) conditions using open-circuit spirometry on VO2000® (VO2000, MedGraphics®□, Saint Paul, Minessota-USA) equipment during the leg ergometer cycle physical test calibrated before each test. The ventilatory equivalent for oxygen (VE/VO_2_), ventilatory equivalent for carbon dioxide (VE/VCO_2_), and respiratory exchange ratio (RER) were recorded at each stage completed to determine maximal oxygen uptake and respiratory exchange quotient. The average of the final two minutes of the test was used to determine the relative VO_2_ [26].

### Outcomes

The main objective and primary outcome of the present study was to evaluate the effect of the formulation on the physical performance of cyclists, in response to this, we evaluated the total exercise time on a leg cycle ergometer, maximum oxygen consumption, mechanical work and mechanical efficiency. The secondary outcomes were the biochemical parameters (NEFA, lactate and RQ) and the rates of perceived exertion using the Borg scale. We obtained the primary and secondary results at the beginning of the test, during and immediately after (20 s) of the progressive load protocol until voluntary fatigue. All tests were performed by the first author, who was unaware of the participants’ allocation.

### Statistical analysis

Data normality was tested using the D’Agostino & Pearson test. The data that were normally distributed were compared using the paired *t*-test. For non-normal data, the Wilcoxon matched pairs signed rank test was used. Information regarding data normality was added to the figures. Data were expressed as mean ± standard deviation (SD) and the significance level was p < 0.05 for all tests. For the evaluation throughout the stages of the physical test, a regression using equations of straight line were also used. To run the test, we chose the fitting method of least squares regression without weighting. The null hypothesis was that one curve would fit the two conditions (HPβ-CD-Placebo and HPβ-CD-Ang-(1–7). The curve comparison method was the extra sum-of-squares F-test and the *p*-value was fixed at 0.05. The measurement of effect size used was Cohen’s d (Cohen’s d = (M2 - M1) ⁄SD_pooled_). The effect size was evaluated based on Cohen’s guidelines: small (0.2), medium (0.5), and large (0.8). The lower 95 % CI of the mean and the upper 95 % CI of the mean for both groups were added to the figure legend.

## Results

### Blood pressure and heart rate responses after HPCD–Ang-(1–7) administered orally

**Fig. 3:**
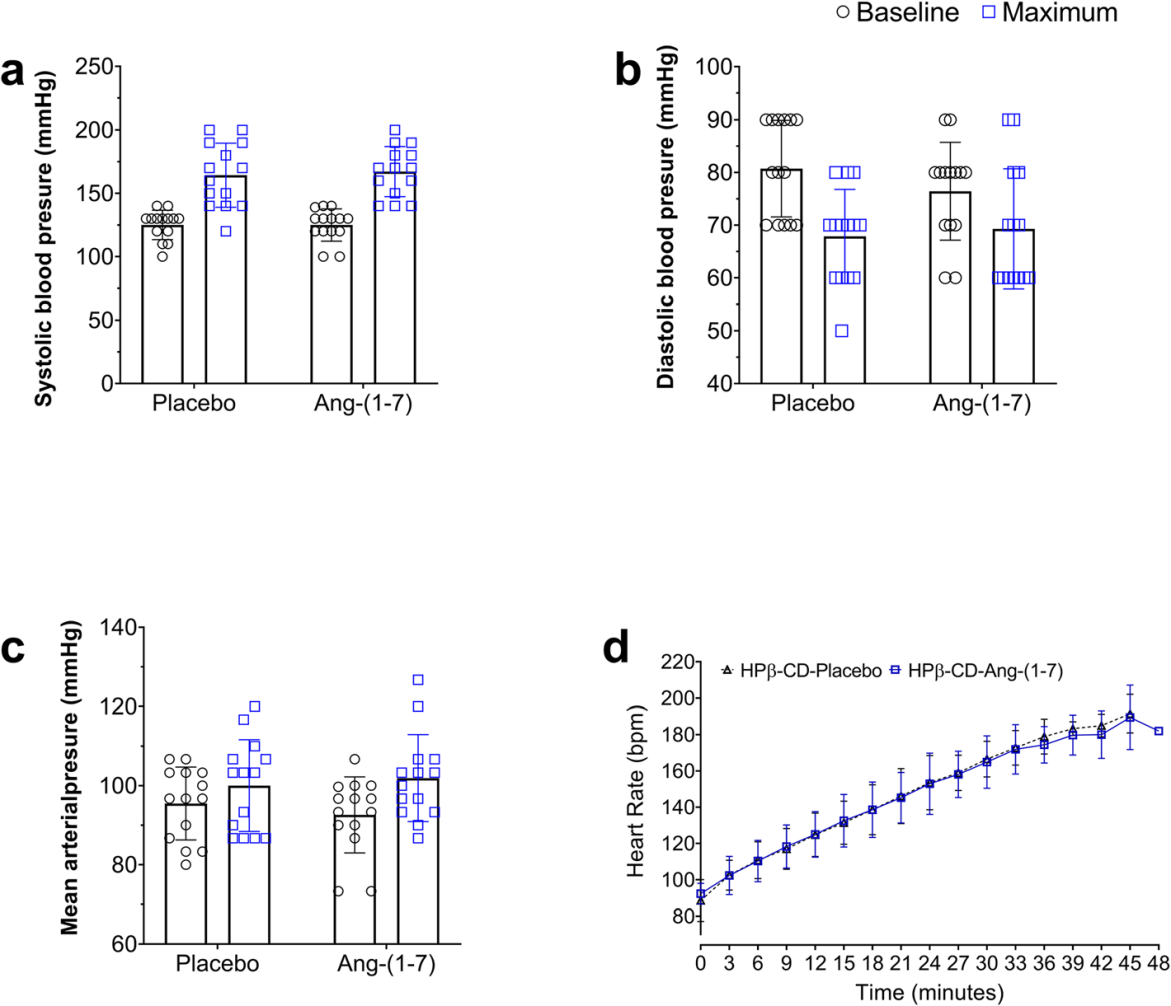
ystolic blood pressure - baseline and maximum (**a**); Diastolic blood pressure - baseline and maximum (**b**); mean arterial pressure - baseline and maximum (**c**); Heart Rate average values throughout the tests (**d**); in HPβ-CD-Placebo and Hβ-CD-Ang-(1-7). Mean ± SD. No differences for parried Systolic blood pressure - baseline and maximum (**a**); Diastolic blood pressure - baseline and maximum (**b**); mean arterial pressure - baseline and maximum (**c**); Heart Rate average values throughout the tests (**d**); in HPβ-CD-Placebo and Hβ-CD-Ang-(1-7). Mean ± SD. No differences for parried. t test overall or Wilcoxon. **a** Baseline - t = 0.01975; Effect Size = ES (Cohen's d = 0.00); Lower 95% CI for Placebo = 116.20 & Ang-(1-7) = 115.60; Upper 95% CI for Placebo = 130.50 & Ang-(1-7) = 131.00 (*n* = 14); **a** Maximum - t = 0.1540; ES, d = 0.03; Lower 95% CI for Placebo = 153.90 & Ang-(1-7) = 156,40; Upper 95% CI for Placebo = 181.50 & Ang-(1-7) = 180.50 (*n* = 14); **b** Baseline - w = -28; ES, d = 0.46; 95% CI of median for HPβ-CD-Placebo = 98.71% & Hβ-CD-Ang-(1-7) = 98.71% (*n* = 14); **b** Maximum - t = 0.4341; ES d = 0.14; Lower 95% CI of mean for HPβ-CD-Placebo = 62.70 & Hβ-CD-Ang-(1-7) = 62.70; Upper 95% CI of mean for HPβ-CD-Placebo = 73.01 & Hβ-CD-Ang-(1-7) = 75.87 (*n* = 14); (c) Baseline - t = 1.024; ES, d = 0.30; Lower 95% CI for Placebo = 90.16 & Ang-(1-7) = 87.04; Upper 95% CI for Placebo = 100.80 & Ang- (1-7) = 98.16 (*n* = 14); **c** Maximum - t = 0.6821; ES, d = 0.17; Lower 95% CI for Placebo = 93.30 & Ang-(1- 7) = 95.56; Upper 95% CI for Placebo = 106.70 & Ang-(1-7) = 108.30 (*n* = 14); **d** No differences between the two conditions HPβ-CD-Placebo and HPβ-CD-Ang-(1-7) during the performance of physical test were observed for the Heart Rate average (F = 1.023; *p* = 0.3604). Mean ± SD in each stage were used to build the graphic (*n* = 14).

Considering the effect of Ang-(1–7) in cardiovascular system, we collected the BP and HR before and after exercise. There were no differences between conditions for SBP in rest (HPβCD-placebo = 123.30 ± 12.9 mmHg vs. HPβCD-Ang-(1–7) = 123.30 ± 13.9 mmHg; or SBP after exercise (HPβCD-placebo = 167.70 ± 22.8 mmHg vs. HPβCD-Ang-(1–7) = 168.50 ± 19.9 mmHg; *p* = 0.8802; Paired t-test) (Fig. 3a).

There were no differences to DBP in rest (HPβCD-placebo = 80.71 ± 9.2 mmHg vs. HPβCD-Ang-(1–7) = 76.43 ± 9.28 mmHg; *p* = 0.1855; Wilcoxon) or after exercise (HPβCD- placebo = 67.86 ± 8.9 mmHg vs. HPβCD-Ang-(1–7) = 69.29 ± 11.4 mmHg; *p* = 0.6714; Paired t-test) (Fig. 3b).

There were no differences between groups for MAP (HPβCD-placebo = 95.48 ± 9.2 mmHg vs. HPβCD-Ang-(1–7) = 92.6 ± 9.6 mmHg; *p* = 0.3246; Paired t-test) or after exercise (HPβCD-placebo = 100.00 ± 11.6 mmHg vs. HPβCD-Ang-(1–7) = 101.90 ± 11.00 mmHg (Fig. 3c).

The HR at rest was as follows: HPβCD-placebo = 58 ± 9.3 bpm vs. HPβCD-Ang-(1–7) = 55 ± 9.0 bpm. In the final of first stage of the test, HR was as follows: HPβCD-placebo = 88 ± 11.5 bpm vs. HPβCD-Ang-(1–7) = 92 ± 5.7 bpm (Fig. 3d). In the final of the last stage, HR was as follows: HPβCD-placebo = 191 ± 10.6 bpm vs. HPβCD-Ang-(1–7) = 189 ± 17.67 bpm (Fig. 3d). No differences for HR were observe between conditions.

### Performance variables after HPCD–Ang-(1–7) administered orally

**Fig. 4:**
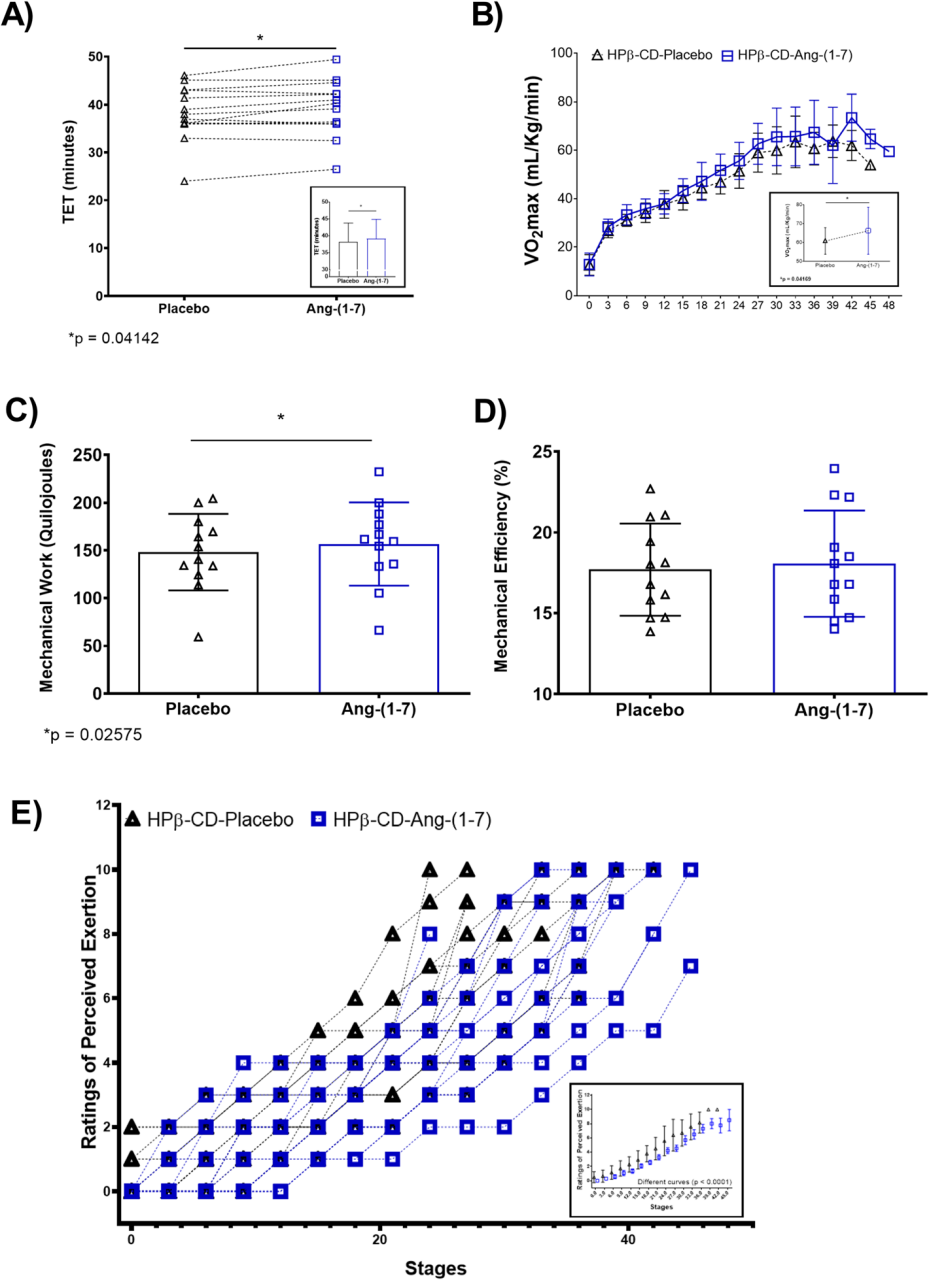
Physical performance. **a** Total exercise time (TET); *p* = 0.04142; *t* = 2.263; Effect Size (ES) (Cohen’s *d* = 0.17); Lower 95 % CI of mean for HPβ-CD-Placebo = 34.88 & Hβ-CD-Ang-(1–7) = 35.76; Upper 95 % CI of mean for HPβ-CD-Placebo = 41.40 & Hβ-CD-Ang-(1–7) = 42.44 (*n* = 14); **b** VO_2_max; *p* = 0.04169; *t* = 2.203; ES *d* = 0.55; Lower 95 % CI for Placebo = 58.07 & Ang-(1–7) = 61.01; Upper 95 % CI for Placebo = 64.57 & Ang-(1–7) = 71.60 (*n* = 14); **c** Mechanical work (MW); *p* = 0.02575; *t* = 2.577; ES *d* = 0.21; Lower 95 % CI for Placebo = 122.6 & Ang-(1–7) = 128.9; Upper 95 % CI for Placebo = 173.7 & Ang-(1–7) = 184.5 (*n* = 12); **d** Mechanical efficiency (ME); *t* = 1.385; ES *d* = 0.10; Lower 95 % CI for Placebo = 15.55 & Ang-(1–7) = 15.63; Upper 95 % CI for Placebo = 18.95 & Ang-(1–7) = 19.47 (*n* = 12); **e** Ratings of Perceived Exertion (RPE); significative difference between the two conditions HPβ-CD-Placebo and HPβ-CD-Ang-(1–7) during the performance of physical test was observed for the RPE (F = 21.77; *p* < 0.0001) box and whisker (min to max)

The total exercise time was significantly higher, approximately one minute, in the treated condition HPβ-CD-Ang-(1–7) = 39.10 ± 5.78 min vs. HPβCD-placebo = 38.14 ± 5.64 min (Fig. 4a), accompanied (as expected) by a higher VO2max: HPβCD-placebo = 60.72 ± 7.03 mlO_2_.kg^− 1^.min^− 1^ vs. HPβCD-Ang-(1–7) = 66.15 ± 12.47 mlO_2_.kg^− 1^.min^− 1^ (Fig. 4b) and a higher level of mechanical work: HPβCD-placebo = 148.6 ± 40.20 kilojoule vs. HPβCD-Ang-(1–7) = 156.7 ± 17.43.71 kilojoule (Fig. 4c). No differences were observed to Mechanical efficiency: HPβCD-placebo = 17.10 ± 2.86 % vs. HPβCD-Ang-(1–7) = 18.07 ± 3.29 % (Fig. 4d).

Interestingly, the linear regression of ratings of perceived exertion were significantly different (p = 0.0001). In this case, individuals who received HPβ-CD-Ang-(1–7) (r² = 0.75) during the performance of physical test reported a lower perception of effort throughout the entire test when compared to HPβCD-placebo (r² = 0.77) and were able to complete the test one stage ahead, at 48 min (Fig. 4e).

### HPCD–Ang-(1–7) administered orally not alter lactate, plasma non non-esterified fatty acids and respiratory exchange coefficient

**Fig. 5:**
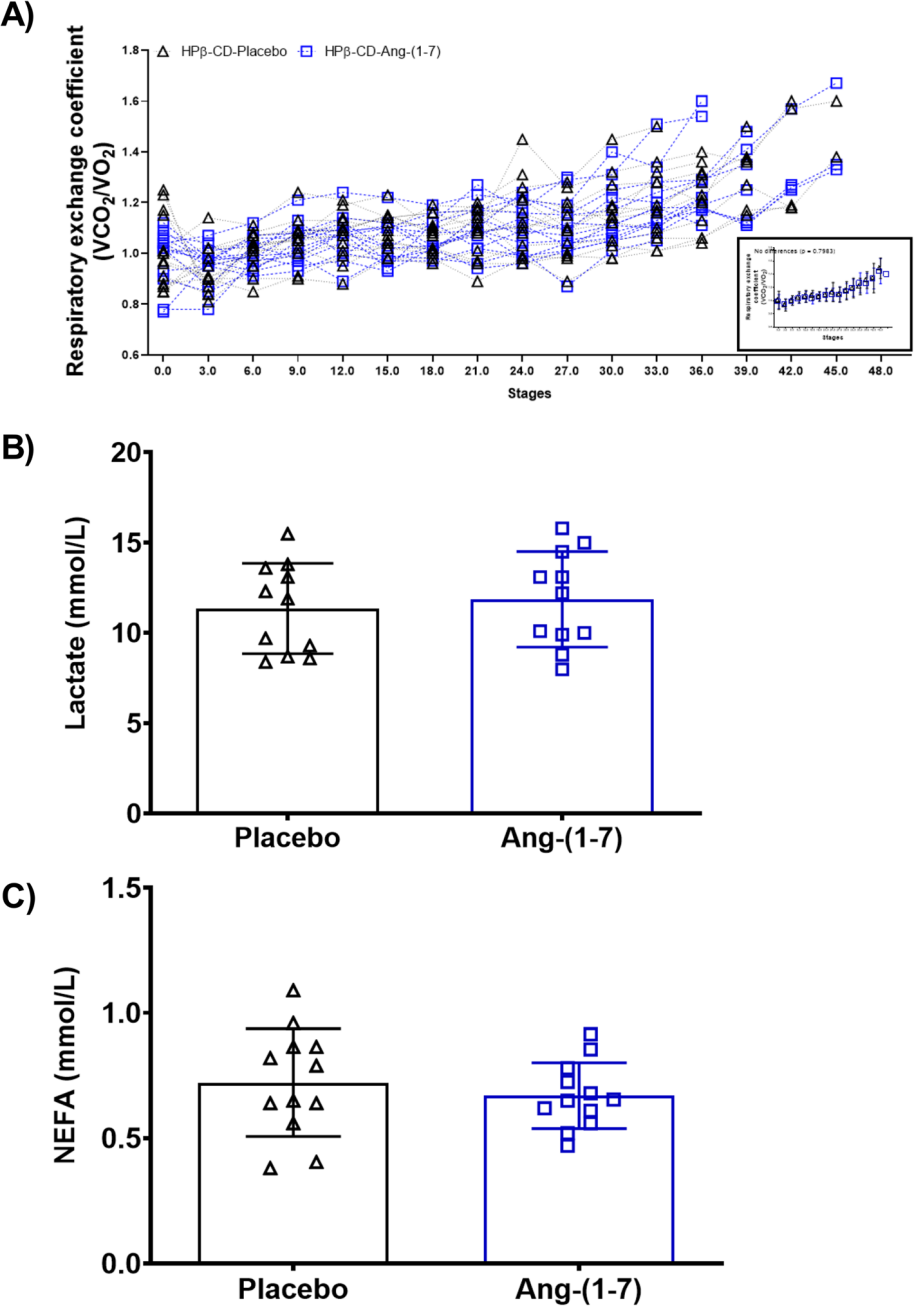
Respiratory exchange coefficient (REC) Lactate and Non-esterified fatty acids (NEFA)**.** **a** There were no differences between the two conditions HPβ-CD-Placebo and HPβ-CD-Ang-(1–7) during the performance of physical test for REC (F = 0.2255; *p* = 0.7983) box and whisker (min to max). **b** Lactate; *t* = 0.4691; ES *d* = 0.20; Lower 95 % CI for Placebo = 9.671 & Ang-(1–7) = 10.08; Upper 95 % CI for Placebo = 13.04 & Ang-(1–7) = 13.64 (*n* = 11); **c** Non-esterified fatty acids (NEFA); *t* = 0.7909; ES *d* = 0.29; Lower 95 % CI for Placebo = 0.5853 & Ang-(1–7) = 0.5863; Upper 95 % CI for Placebo = 0.8588 & Ang-(1–7) = 0.7537 (*n* = 12); Mean ± SD (parried *t* test)

Considering the effects of Ang-(1–7) on metabolic control, we found that the respiratory exchange coefficients were not significantly different (p = 0.36) when comparing HPβCD-placebo (r² = 0.81) vs. HPβCD-Ang-(1–7) (r² = 0.85) (Fig. 5a). Note that the columns almost overlap and that a single line explains the behavior of the two groups for respiratory exchange coefficient (Fig. 4a).

The lactate levels HPβCD-placebo = 11.35 ± 2.50 mmol/L vs. HPβCD-Ang-(1–7) = 11.86 ± 2.65 mmol/L (Fig. 5b) and the concentration of non-esterified fatty acids (NEFA) HPβCD-placebo = 0.72 ± 0.21mmol/L vs. HPβCD-Ang-(1–7) = 18.07 ± 3.29 mmol/L (Fig. 5c) not were different between conditions.

## Discussion

The present study is the first to show that there is an effect of using an oral formulation of HPβ-CD-Ang-(1–7) with relatively low doses of the peptide (1.75 mg) on the physical performance of MTB athletes. The results show a significant difference in TET, MW, maximal oxygen uptake, and lower RPE.

The oral formulation HPβ-CD-Ang-(1–7) was not associated with cardiovascular alterations because there were no differences in HR and BP between conditions at rest or after strenuous exercise compared to the placebo, suggesting that this dose do not affect cardiovascular system. Traditionally the Ang-(1–7) is associated to cardiovascular effects, so this present work is the first that observed the cardiovascular response induced by the Ang-(1–7) associates with exercise in humans and more specific in this case, in athletes.

Clinical studies in healthy subjects, show that the Ang-(1–7) has no effect in blood pressure [27, 28]. The dose used in the present study is low compared to the study that evaluated antiangiogenic effect of Ang-(1–7), the maximum dose that produces toxicity was 700 µg/kg [23], our study used a dose sixteen time lower.

It is well established that performance in aerobic endurance exercise is related to maximal oxygen uptake (VO_2_max), mechanical economy/efficiency during exercise and lactate threshold [29]. In the present study, the athlete under the influence of the oral formulation HPβ-CD-Ang-(1–7) increased the total effort time, mechanical work and consumed an average of 6mlO_2_.kg^− 1^.min^− 1^ more oxygen at peak exercise effort. The possible mechanisms involved in increased physical performance may involve effects of Ang-(1–7) such as vasodilation, increased blood flow.

The vasodilatory effects of Ang-(1–7) were observe in arterioles of the adipose and atrial tissue of patients with or without clinical diagnosis of coronary artery diseases. [30] Intra-brachial infusion of the Ang-(1–7) increased forearm blood flow in healthy and hypertensive subjects [31], reduces Ang II-induced vasoconstriction in mammary arteries of healthy subjects [32]. Ang-(1–7) stimulated the production of endothelium-derived nitric oxide, prostaglandins, and relaxation factors in endothelial cells in animal models [33]. Acute infusion of Ang-(1–7) led to significant changes in blood flow distribution and decreased in total peripheral resistance [34]. Similarly, in another study of transgenic mice [35] expressing an Ang-(1–7) producing fusion protein, there was a reduction in total peripheral resistance, suggesting that the acute increase in Ang-(1–7) may lead to important regional and systemic hemodynamic changes.

These data strongly suggest that Ang-(1–7) may recruit muscle microvasculature and increase the area of the microvascular endothelial surface, leading to increased nutrient and oxygen delivery to the skeletal musculature [14]. Our hypothesis was that the Ang-(1–7) would increase vasodilation and blood flow, thereby improving VO_2_max by augmenting the nutrient and oxygen delivery to skeletal muscle.

One interesting date and that our first time observed in humans, was the lower rates of perceived exertion when the athletes were supplement by HPβ-CD-Ang-(1–7). The higher central levels of monoamines as well NE are associated with increase in central fatigue during exercise [18]. Date in literature in animal model describe that the Ang-(1–7) via the Mas receptor, promote an increase on neuronal uptake, and modulating a decrease in the release and synthesis of NE, through a mechanism mediated by BK / NO [36, 37].

Recent data from our group show that transgenic rats overexpressing circulating Ang-(1–7), when subjected to strenuous exercise (6 h), had lower plasma glucose variations, lower hepatic, and muscle glycogen depletion [38]. Another study [14] demonstrated increased muscle microvascular recruitment following Ang-(1–7) infusion, increasing glucose uptake via the Glut-4 receptor.

These findings suggest that administration of Ang-(1–7) improves glucose metabolism both at rest and during exercise. Our work measure RER, lactate and NEFA in plasma with aim to found out the effects of formulation in metabolic via used during exercise, but the date does not show differences.

The limitations of present study where we do not measure the glucose before and after exercise, probably the protocol used in this study has a short time to induced significant glucose alterations, the animals study cited above use an extensive protocol, mean 6 h of the exercise strenuous. Future studies will be necessary in athletes submitted to long exercise protocols associated to supplementation with Ang-(1–7), maybe this type of exercise can be showing several factors, including the glucose alterations and other important parameters for physical performance.

World MTB competitions are decided by a difference of seconds. Therefore, the present study provides evidence that acute supplementation with the formulation of HPβ-CD-Ang-(1–7) may be potential to achieve decisive results in this modality.

## Conclusions

The oral formulation of HPβ-CD-Angiotensin-(1–7) (1.75 mg) improves the physical performance of MTB athletes. There were increases in TET and MW, as well as higher oxygen consumption and lower RPE. There was no difference in cardiovascular parameters between the placebo and the treated condition at rest or at peak physical effort.

### Future studies

The mechanisms involved in increase in physical performance describe above as vasodilatory and glucose uptake need to be investigated, local analyses in specific tissues as well skeletal muscle must be analyzed: for example, muscle biopsy and regional blood flow by Color Doppler Echocardiography.

## Data Availability

The datasets generated and/or analyzed as part of the current study are not publicly available due to confidentiality agreements with subjects. However, they can be made available solely for the purpose of review and not for the purpose of publication from the corresponding author upon reasonable request.

## Abbreviations

MTB: MTB
HR: Heart rate
BP: Blood pressure
VO2: Maximum oxygen consumption
ETT: Total exercise time
MW: Mechanical work
ME: Mechanical efficiency
RPE: Perceived effort
REC: Respiratory exchange coefficient
NEFAs: Unesterified fatty acids
CK: Creatine kinase
M: Mean
SD: Standard deviation
SBP: Systolic blood pressure
DBP: Diastolic blood pressure
MAP: Mean arterial pressure
